# The Utility of the Platelet-Albumin-Bilirubin Score as a Non-invasive Predictor of Esophageal Varices and Variceal Hemorrhage in Patients With Liver Cirrhosis Compared to Child-Turcotte-Pugh and Model of End-Stage Liver Disease-Sodium Scores

**DOI:** 10.7759/cureus.62577

**Published:** 2024-06-18

**Authors:** Ayesha Malik, Mahrosh Asif, Rafi Ud Din, Asma Khan, Muhammad Siddique, FNU Noor, Hala Mansoor, Aamir Habib

**Affiliations:** 1 Gastroenterology and Hepatology, Combined Military Hospital, Lahore, PAK; 2 Medicine, Combined Military Hospital, Lahore, PAK

**Keywords:** upper gastro-intestinal bleed, platelet-albumin-bilirubin score, meld-na, esophageal and gastric varices, cirrhosis liver, child-pugh score

## Abstract

Introduction

Research on non-invasive tools for detecting gastro-esophageal varices is underway. We investigated the Platelet-Albumin-Bilirubin (PALBI) score in comparison with the Child-Turcotte-Pugh (CTP) and MELD-Na (MELD-Na) scores in patients with liver cirrhosis.

Methods

Three hundred and twenty-three patients with liver cirrhosis were studied. The PALBI, CTP and MELD-Na scores were calculated and analyzed for gastroesophageal varices and their characteristics using SPSS version 26 (IBM Corp., Armonk, NY, USA).

Results

Two hundred and sixty-four patients had esophageal varices and 102 presented with variceal hemorrhage. Mean PALBI, CTP and MELD-Na scores were significantly higher for patients with varices versus without varices (p < 0.05). Unlike the mean MELD-Na score, the mean PALBI and CTP scores were significantly higher in patients with large high-risk varices as compared to patients with small low-risk varices (p < 0.05). The mean CTP scores were significantly higher in patients with variceal hemorrhage than those without hemorrhage (p < 0.05), while the difference between mean PALBI and MELD-Na was insignificant, in this regard. The PALBI score had better sensitivity than the CTP and MELD-Na scores in indicating the presence of varices but was similar to the CTP score in predicting high-risk varices.

Conclusion

The PALBI score proves to have good utility and efficiency in predicting varices in comparison to CTP and MELD-Na scores. It can determine high-risk stigmata of variceal hemorrhage with similar performance as the CTP Score.

## Introduction

Variceal hemorrhage is one of the leading causes of liver cirrhosis-related morbidity, having a six-week post-hemorrhage mortality rate as high as 20% [1-3]. Variceal hemorrhage is preceded by a series of pathogenic mechanisms which emerge once fibrotic scar tissue accumulates in the cirrhotic liver [4]. Fibrosis leads to structural changes in the hepatic sinusoids which increases resistance to portal flow. As the portal pressure increases it leads to portal hypertension, defined as a hepatic venous pressure gradient (HVPG) of more than 5 mmHg [5,6]. When the pressure rises even further, the blood flows into porto-systemic collaterals, decompressing the hypertensive portal vein while forming gastroesophageal varices [7]. When the HVPG rises beyond 12 mmHg, the variceal wall ruptures presenting as a life-threatening hemorrhage [8].

Timely diagnosis of gastro-esophageal varices is vital to prevent upper gastrointestinal (GI) bleed and upper GI endoscopy is the gold standard for diagnosis and treatment [9,10]. In specialized centers, upper GI endoscopies are routinely performed for screening purposes and post-hemorrhage therapeutic management once liver cirrhosis or portal hypertension is established. In resource-poor areas, the use of screening endoscopies to detect silent varices must be rationalized. It is an invasive and expensive test which many patients are reluctant to undergo, especially in these areas where only a pharyngeal throat spray substitutes conscious sedation [11].

The need to identify sensitive and specific non-invasive markers of esophageal varices and hemorrhage has been a long-standing area of interest. Various clinical and biochemical parameters are currently being researched for their utility in assessing the need for endoscopic variceal screening. Among these parameters are the Child-Turcotte-Pugh (CTP) score, Model for End-Stage Liver Disease (MELD-Na) score, Aspartate Aminotransferase (AST) to Platelets Ratio (APRI score), Baveno VI criteria, and platelet count/spleen diameter ratio. Many studies support their use as predictors of esophageal varices [12,13]. The well-validated Baveno VI criteria is another accurate predictor for the risk of varices which combines the use of transient elastography (TE) and platelet count, but may not be a standard tool as TE is not readily available [14].

Recently, a novel Albumin-Bilirubin (ALBI) score was developed to determine prognosis in patients with hepatocellular carcinoma undergoing resection. Roayaie et al. [15] modified this score by adding platelet count and forming the Platelet-Albumin-Bilirubin (PALBI) score. Our study aims to evaluate the reliability and diagnostic value of the PALBI score, in comparison to CTP and MELD-Na scores, as a non-invasive sensitive marker of esophageal varices and variceal bleed in patients with liver cirrhosis in Pakistan.

## Materials and methods

Study design

A single-center, prospective, cross-sectional study was conducted at the Department of Gastroenterology and Hepatology of Combined Military Hospital Lahore, Pakistan. The hospital’s ethical review board approved this study. All participants signed an informed consent.

Data collection

Patients with chronic liver disease presenting consecutively to the outpatient clinic or emergency suite over a period of six months having clinically significant portal hypertension, with or without upper GI bleed, were all recruited. Cirrhosis was confirmed in 377 patients using ultrasound or transient elastography, in conjunction with clinical and biochemical parameters. Cirrhotic patients regardless of etiology were all included in the study. Patients diagnosed with hepatocellular carcinoma, portal vein thrombosis, patients taking beta blockers, having incomplete laboratory investigations, positive history for prior endoscopy, history of variceal band ligation or previous upper GI bleed, and comorbid chronic kidney disease or hematological disorders - conditions affecting the MELD-Na or PALBI score - were excluded from the study.

All patients underwent standard biochemical investigations including complete blood picture, liver and renal function tests, international normalized ratio (INR) and serum electrolytes. Detailed histories and a thorough physical examination were completed. Upper GI endoscopies were performed by two designated endoscopists. The presence or absence of esophageal and gastric varices along with their size was documented. The size of varices was detailed as per the Baveno classification into Grade I, II and III. The presence of high-risk stigmata of bleed such as cherry red spots and red “wale” marks were also noted. A diagnosis of variceal hemorrhage was confirmed when actively oozing or spurting varices were observed. When the varices were not actively bleeding but had high-risk stigmata or signs of recent hemorrhage, in the absence of any other bleeding lesion, variceal hemorrhage was inferred.

PALBI score was calculated using the following equation:

PALBI = 2.02 × log_10_ TBIL − 0.37 × (log_10_ TBIL)^2^ − 0.04 × ALB− 3.48 × log_10_ PLT+ 1.01 × (log_10_ PLT)

where TBIL is bilirubin (μmol/L), ALB is albumin (g/L) and PLT is platelet (10^9^/L).

The Child-Turcotte-Pugh score was calculated using bilirubin (mg/dL), albumin (g/dL), INR, ascites, and hepatic encephalopathy parameters [16]. MELD-Na score was calculated using the Model of End-Stage Liver Disease (MELD) Score as shown below:

MELD = 9.57 × ln (Cr) + 3.78 × ln (TBIL) + 11.2 × ln (INR) + 6.43

MELD-Na = MELD + 1.32 × (137 - Na) - [0.033 × MELD (137 - Na)]

where Cr is serum creatinine and Na denotes sodium level (mEq/L).

Statistical analysis

Data was tabulated and analyzed using IBM SPSS Statistics for Windows, Version 26.0 (Released 2019; IBM Corp., Armonk, NY, USA). Continuous variables were expressed as means ± standard deviation (SD) while non-normally distributed data were expressed as medians and interquartile ranges (IQR). Categorical data were presented as frequencies and percentages. Student’s t-test was used to compare means of normally distributed data while the Mann-Whitney U test was used to compare mean ranks for non-normally distributed data. One-way ANOVA and Kruskal-Wallis test were used to assess the significance between groups for normally and non-normally distributed data respectively. P value <0.05 with a confidence interval of 95% was considered statistically significant. Univariate and multivariate analyses were done. Receiver Operator Characteristic (ROC) Curves were plotted to compare the efficiency of the scores and determine the positive and negative predictive values.

## Results

A total of 323 patients met the eligibility criteria after excluding 51 patients. There were 192 (59.4%) male participants and 131 (40.6%) female participants. The median age of the study group was 57 (11) years. Viral hepatitis C was the causative factor of liver cirrhosis for 262 (81.1%) patients. Other etiologies included viral hepatitis B, hepatitis B/C co-infection, non-alcoholic liver cirrhosis, autoimmune hepatitis and alcoholic liver cirrhosis. One hundred and two (31.6%) out of 323 patients presented with variceal hemorrhage.

Two hundred and sixty-four (81.7%) patients presented with esophageal varices while 34 (10.5%) patients had gastric varices. Cases of gastric variceal bleed (four patients) were excluded from the analysis.

**Figure 1:**
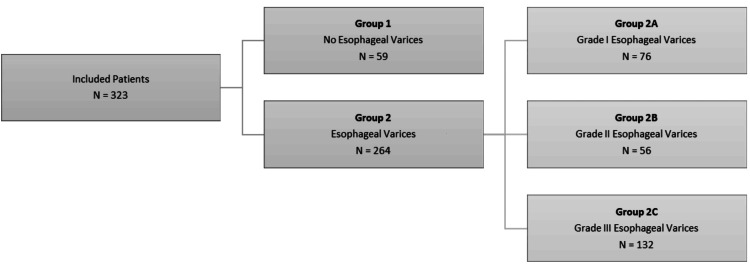
Groups and subgroups of patients with liver cirrhosis N: number of patients

Figure 1 shows the patient groups based on the upper GI endoscopy diagnosis of esophageal varices and subgroups as per Baveno grading criteria.

**Figure 2:**
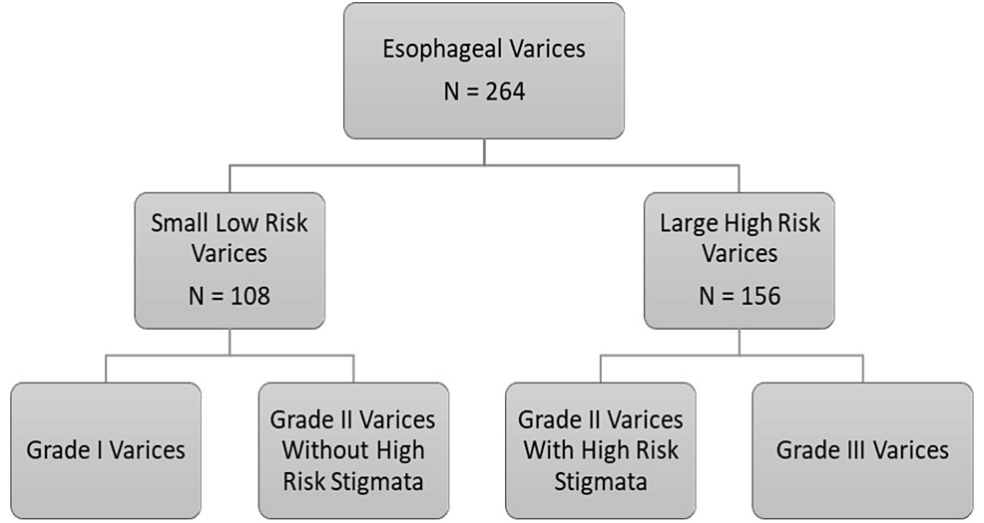
Categories based on variceal size and high-risk endoscopic stigmata N: number of patients

Figure 2 represents categories based on size and endoscopic stigmata for risk of variceal hemorrhage.

According to Table 1, those with esophageal varices had a statistically significantly higher mean PALBI score than those without varices (p = 0.000). Similarly, they had a significantly higher CTP score (U=3883, p=0.000) and MELD-Na score (U=6255, p=0.018).

**Table 1:** Comparison of mean scores between Group 1 (No Varices) and Group 2 (Varices) p-value is significant for <0.05 PALBI: Platelet-Albumin-Bilirubin Score, CTP: Child-Turcotte-Pugh Score, MELD-Na: Model of End-Stage Liver Disease-Sodium Score, N: Number of patients.

Groups	PALBI Score	CTP Score	MELD-Na Score
Group 1 (N = 59)	-2.73 ± 0.30	6.22 ± 1.48	11.36 ± 4.60
Group 2 (N = 264)	-2.21 ± 0.44	7.77 ± 1.83	13.08 ± 5.46
p-value	0.000	0.000	0.018

Post hoc analysis for subgroups depicted that the mean PALBI score was significantly lower in group 2A than both group 2B and 2C (p = 0.000). There was no statistically significant difference between mean PALBI scores for group 2B and group 2C. Group 2A had statistically significantly lower mean CTP scores than both group 2B and 2C (p = 0.000). Group 2B had statistically significantly lower mean CTP scores than group 2C (p = 0.000). The mean MELD-Na score was not statistically significant when compared among subgroups.

As demonstrated in Table 2, CTP and PALBI scores were statistically significantly higher for patients with high-risk varices in comparison to those with low-risk varices (p=0.000).

**Table 2:** Comparison of scores for risk categories of varices and variceal hemorrhage p-value is significant for <0.05 PALBI: Platelet-Albumin-Bilirubin Score, CTP: Child-Turcotte-Pugh Score, MELD-Na: Model of End-Stage Liver Disease-Sodium Score, N: Number of patients.

		PALBI Score	CTP Score	MELD-Na Score
Risk Categories	Small Low-Risk Varices (N = 108)	-2.39 ± 0.39	6.84 ± 1.57	12.86 ± 5.63
	Large High-Risk Varices (N = 156)	-2.09 ± 0.43	8.41 ± 1.72	13.24 ± 5.36
	p-value	0.000	0.000	0.359
Variceal Hemorrhage	Yes (N = 102)	-2.25 ± 0.43	8.11 ± 1.84	12.62 ± 5.22
	No (N = 162)	-2.16 ± 0.45	7.56 ± 1.80	13.38 ± 5.61
	p-value	0.119	0.024	0.327

The mean CTP scores were significantly higher in patients presenting with variceal hemorrhage, as shown in Table 2. There was no difference in mean PALBI and MELD-Na scores between the hemorrhage and no hemorrhage groups. The CTP, PALBI and MELD-Na scores were similar for patients with and without gastric varices.

**Figure 3:**
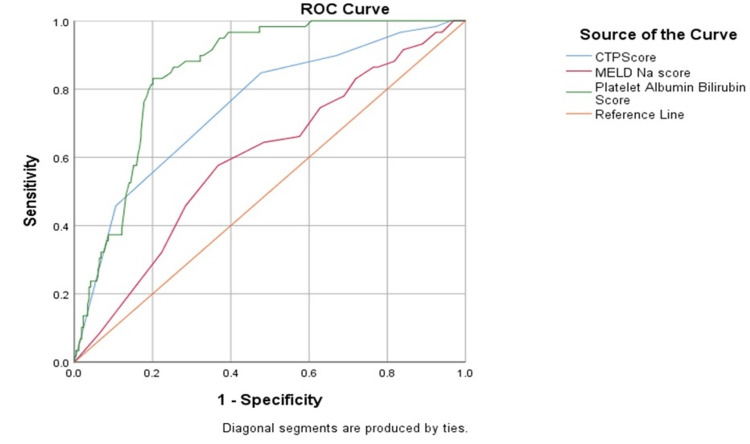
Receiver Operator Curve (ROC) of each score distinguishing Group 1 from Group 2

Receiver operator characteristic curves (ROC) comparing the predictive power of PALBI, CTP and MELD-Na scores for the presence of esophageal varices showed the PALBI score to have a high sensitivity and high positive predictive value for a cut-off of -2.58 (Figure 3) (Table 3).

**Table 3:** Receiver Operator Characteristic (ROC) analysis of each score for Group 1 vs Group 2 p-value is significant for <0.05 PALBI: Platelet-Albumin-Bilirubin Score, CTP: Child-Turcotte-Pugh Score, MELD-Na: Model of End-Stage Liver Disease-Sodium Score, AUC: Area under the curve, PPV: Positive predictive value, NPV: Negative predictive value.

Score	Cut off	AUC	Sensitivity (%)	Specificity (%)	PPV	NPV	p-value
CTP	6.50	0.75	73.1	62.7	0.90	0.34	0.000
MELD-Na	9.50	0.59	71.6	45.8	0.86	0.29	0.018
PALBI	-2.58	0.85	83.0	67.8	0.92	0.47	0.022

**Figure 4:**
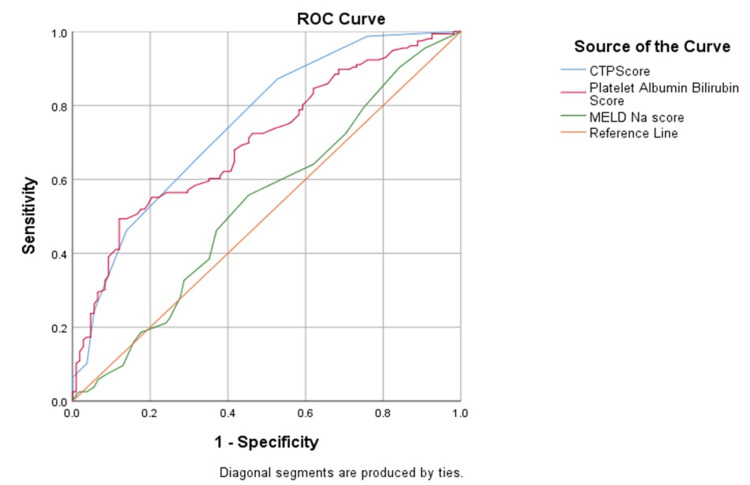
Receiver Operator Curve (ROC) of each score for large high-risk varices

Both CTP and PALBI scores have a similar sensitivity and accuracy for a cut-off value of 7.50 and -2.27 respectively in identifying high-risk varices, shown in Figure 4 and Table 4. The accuracy of MELD-Na score was insignificant in this regard (p > 0.05). CTP and PALBI scores have high positive predictive values.

**Table 4:** Receiver Operator Characteristic (ROC) analysis of each score for large high-risk varices p-value is significant for <0.05 PALBI: Platelet-Albumin-Bilirubin Score, CTP: Child-Turcotte-Pugh Score, MELD-Na: Model of End-Stage Liver Disease-Sodium Score, AUC: Area under the curve, PPV: Positive predictive value, NPV: Negative predictive value.

Score	Cut off	AUC	Sensitivity (%)	Specificity (%)	PPV	NPV	p-value
CTP	7.50	0.75	66.0	67.6	0.75	0.58	0.000
MELD-Na	10.50	0.53	64.1	38.0	0.60	0.42	0.361
PALBI	-2.27	0.71	62.2	61.1	0.70	0.53	0.000

**Figure 5:**
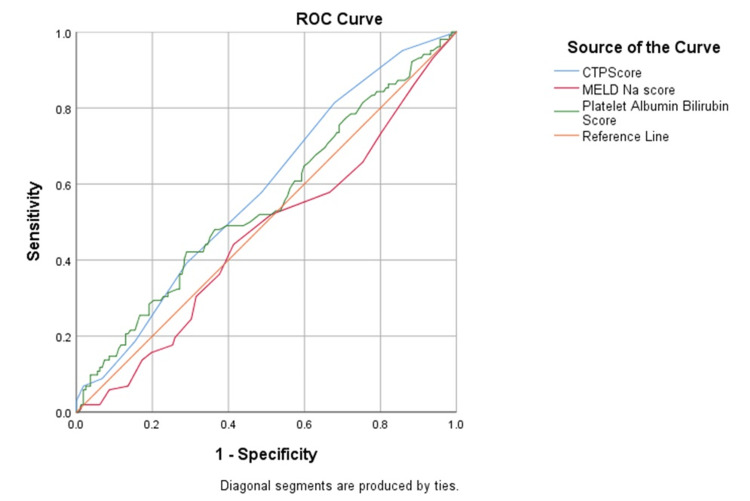
Receiver Operator Curve (ROC) of each score for variceal hemorrhage

The ROC model for an outcome of variceal hemorrhage showed low sensitivity and specificity for the CTP score for a cut-off value of 7.50, while the PALBI and MELD-Na scores proved to be insignificant in accurately determining variceal bleed (Figure 5 and Table 5). None of the three scores proved to be significant in predicting gastric varices (p > 0.05).

**Table 5:** Receiver Operator Characteristic (ROC) analysis of each score for variceal hemorrhage p-value is significant for <0.05 PALBI: Platelet-Albumin-Bilirubin Score, CTP: Child-Turcotte-Pugh Score, MELD-Na: Model of End-Stage Liver Disease-Sodium Score, AUC: Area under the curve, PPV: Positive predictive value, NPV: Negative predictive value.

Score	Cut off	AUC	Sensitivity (%)	Specificity (%)	PPV	NPV	p-value
CTP	7.50	0.61	58.0	51.2	0.68	0.41	0.026
MELD-Na	9.50	0.46	65.7	24.7	0.66	0.43	0.328
PALBI	-2.50	0.56	81.4	25.0	0.61	0.30	0.165

On multivariate analysis, neither the age nor the gender and etiology of cirrhosis were independent predictors of the presence of varices or their size. The presence of ascites was positively correlated with the presence of varices and high-grade varices as compared to low-grade varices (p=0.000).

## Discussion

Gastro-esophageal varices signal the onset of one of the most sinister outcomes of decompensated chronic liver disease. Approximately 50% of all patients with liver cirrhosis have esophageal varices and the risk of bleed increases with the severity of liver disease [17,18]. Several studies have documented the use of non-invasive tests for screening of varices prior to endoscopy but the majority of tests were found to be unreliable predictors [19]. We found the PALBI score to be a simple, inexpensive and reliable potential predictive test which could help triage patients prior to upper GI endoscopy in the future.

The PALBI score accurately corresponded with endoscopy findings in predicting the presence of esophageal varices. It was both sensitive and had a high diagnostic accuracy in this regard. The mean PALBI scores were significantly higher in patients with esophageal varices and high-risk types. This test was initially used to predict the outcomes of interventional management of hepatocellular carcinoma [20]. Although it is thought to reflect portal hypertension more accurately, due to the inclusion of the platelet count, few studies have researched the PALBI score as a marker of varices [21]. Our findings signal a promising future for its use as a standard non-invasive predictor of varices when compared to the CTP and MELD-Na scores.

The Child-Pugh (CTP) score is a widely used scoring system which assesses the severity of liver disease. Those with advanced liver disease and high scores tend to present with large high-risk varices and a greater risk of first variceal hemorrhage according to the North Italian Endoscopic Club (NIEC) Index and the Rev-NIEC (Revised NIEC) [22]. Inspiringly, we found the PALBI score to have a better sensitivity than the CTP score in indicating the presence of esophageal varices [19]. The PALBI score has the benefit of including the bilirubin and albumin levels, similar to the CTP score, while excluding potentially confounding and subjective parameters such as ascites and hepatic encephalopathy. Both these scores had similarly good sensitivities and positive predictive values for high-risk varices. However, the CTP score had a better performance than the PALBI score in predicting variceal hemorrhage as compared to the PALBI score.

The MELD-Na score has replaced the MELD score as a superior prognostic tool for cirrhotic patients. Since high MELD scores were often reported as a potential risk factor for variceal hemorrhage, this necessitated the need to evaluate the role of MELD-Na in this regard [23,24]. Consequently, it was proved that the MELD-Na score is a good predictor for the presence of esophageal varices [25]. In our population, the mean MELD-Na score was remarkably higher in those with esophageal varices in comparison to those without varices. However, there was no significant difference in mean MELD-Na scores between the high and low-risk varices group or between variceal bleed and no bleed. The other two scores performed better than the MELD-Na score on ROC analysis giving this score lesser value as a predictive tool.

Contrary to our findings, Glisic et al. proved the MELD score to have the best performance as a predictive test for varices amongst all known non-invasive scores. The Siberian study also concluded that the PALBI score has some value in predicting the presence of varices, variceal hemorrhage and mortality [26]. The difference in our results can be attributed to the variations in etiology as the majority of the patients in the Siberian population had alcoholic liver cirrhosis. In our population, viral hepatitis C was the most common cause of liver cirrhosis [27].

This study is one of the few studies that have investigated the PALBI score for varices, their characteristics, and variceal bleed. Large-scale studies have previously found the PALBI score to be a superior prognostic indicator, although after variceal hemorrhage, in comparison to the CTP, MELD and ALBI scores [28]. Patients with higher PALBI scores had higher mortality and were more likely to re-bleed after therapeutic intervention via upper GI endoscopy [29,30]. We compared scores prior to a variceal hemorrhage and found significantly higher PALBI scores in patients who had high-risk stigmata. Further research and stratification of the PALBI score in correlation to the risk of variceal hemorrhage can enhance its utility in determining patient outcomes in cirrhosis.

Our study had some limitations. This was a single-center study conducted on a limited population of patients. Some patients presenting to the department had gastric variceal hemorrhage. The utility of the scores could not be determined for these patients owing to the small patient population. Further large-scale, multi-centric studies are needed to find the role of these scores in predicting gastric varices and gastric variceal hemorrhage. A comparison between the scores for gastric compared to esophageal varices can also help discover unknown specific attributes of each score.

## Conclusions

The PALBI score is a useful tool in predicting the presence of varices and high-risk stigmata of variceal hemorrhage. It has a relatively better performance than the MELD-Na score and an analogous performance to the CTP score as a predictive tool. In some instances, the PALBI score has superior value to both scores such as predicting esophageal varices. The PALBI failed to be of benefit in predicting index variceal hemorrhage. None of the scores have any value for patients with gastric varices.
